# Advancements in lipid production research using the koji-mold *Aspergillus oryzae* and future outlook

**DOI:** 10.3389/ffunb.2024.1526568

**Published:** 2024-12-16

**Authors:** Koichi Tamano

**Affiliations:** ^1^ Bioproduction Research Institute, National Institute of Advanced Industrial Science and Technology (AIST), Sapporo, Japan; ^2^ Computational Bio Big-Data Open Innovation Laboratory (CBBD-OIL), National Institute of Advanced Industrial Science and Technology (AIST), Tokyo, Japan

**Keywords:** Aspergillus oryzae, filamentous fungus, lipid production, free fatty acid, ester-type fatty acid, metabolic improvement, gene knockout, gene overexpression

## Abstract

Research on enhancing the production of lipids, particularly polyunsaturated fatty acids that are considered important for health, has focused on improvement of metabolism as well as heterologous expression of biosynthetic genes in the oleaginous fungus *Aspergillus oryzae*. To date, the productivity and production yield of free fatty acids have been enhanced by 10-fold to 90-fold via improvements in metabolism and optimization of culture conditions. Moreover, the productivity of ester-type fatty acids present in triacylglycerols could be enhanced via metabolic improvement. Culturing *A. oryzae* in a liquid medium supplemented with non-ionic surfactants could also lead to the effective release of free fatty acids from the cells. The current review highlights the advancements made in this field so far and discusses the future outlook for research on lipid production using *A. oryzae*. I hope the contents are useful for researchers in this field to consider the strategy of increasing production of various valuable metabolites as well as lipids in *A. oryzae*.

## 1 Introduction

*Aspergillus oryzae* is a filamentous fungus used in the manufacturing of numerous fermented foods. One of the superior characteristics of *A. oryzae* is its high capacity to produce biomolecules including hydrolytic enzymes. Utilizing this characteristic, amylases that degrade the starch in rice are used to manufacture rice wine (sake), whereas proteases that degrade the protein in soybeans are used to manufacture soy sauce (shoyu) and soybean paste (miso). Moreover, *A. oryzae* has been scientifically certified as safe for food fermentation because it does not produce bioactive compounds that interfere with human health (Machida et al., 2005; Payne et al., 2006). Therefore, *A. oryzae* is generally recognized as safe (GRAS) by the United States Department of Agriculture and is internationally regarded as edible.

The whole genome of *A. oryzae* wild-type strain RIB40 was first sequenced in 2005 (Machida et al., 2005), enabling research based on genomic information (Abe et al., 2006). Also, various genetic engineering techniques and genetic tools have been developed, including gene targeting (Takahashi et al., 2006; Mizutani et al., 2008; Zhang et al., 2017a), selectable markers (Jin et al., 2021), expression promoters (Jin et al., 2021), and genome editing (Maruyama, 2021; Jin et al., 2022; Tamano, 2022). These advancements now allow for the knockout or overexpression of target genes (Jin et al., 2021) and the introduction of long DNA fragments from other species into specific genomic loci (Yoshimi et al., 2018).

*A. oryzae* is also an oleaginous fungus, characterized by having over 20% lipids in its dry cell weight (Hassane et al., 2024). Other oleaginous fungi include *Mortierella* spp., *Mucor* spp., and *Aspergillus* spp. Notably, *Mortierella* spp. and *Mucor* spp. possess high lipid content and produce polyunsaturated fatty acids (PUFAs), making them valuable for industrial PUFA production and beneficial to human health (Mohamed et al., 2020; Fazili et al., 2022; Zhang et al., 2022). While *A. oryzae* has lower lipid levels than *Mortierella* spp., it still contains a relatively high amount of lipids (Meng et al., 2009). Additionally, due to its low secondary metabolic activity, *A. oryzae* produces few secondary metabolites (Frisvad et al., 2018; Tamano et al., 2019), making it an ideal host for the heterologous expression of valuable secondary metabolites (Nagamine et al., 2019; Qi et al., 2022; Han et al., 2023).

If oleaginous fungi used in lipid production produce valuable lipids, they can be used in the wild-type form. For instance, *Mortierella alpina* produces arachidonic acid as PUFA, which is beneficial for health; therefore, the wild-type strain can be industrially used (Bajpai et al., 1991). Also, a spontaneous mutant of *M. alpina* lacking the Δ5-desaturase gene accumulates dihomo-γ-linolenic acid as the final product (Jareonkitmongkol et al., 1993). Given that dihomo-γ-linolenic acid is a precursor of prostaglandin E1 that is used as a pharmaceutical agent, the mutant is deemed useful as the supplier of feedstock for biosynthesis. Additionally, *Mucor circinelloides* and *Mucor plumbeus* originally produce γ-linolenic acid, which serves as a nutritional supplement, enhancing its industrial utility (Fazili et al., 2022; Mohamed et al., 2022a, b).

In contrast, *A. oryzae* produces four fatty acids (FAs): palmitic acid, stearic acid, oleic acid, and linoleic acid (Tamano et al., 2015). They are different from PUFA, and thus, the wild-type *A. oryzae* seems inappropriate for lipid production. In such cases, introducing functional genes involved in FA polyunsaturation derived from other organisms into *A. oryzae* is necessary to make it produce PUFA.

In this mini review, I focus on *A. oryzae* in oleaginous fungi, summarize the lipid production research using *A. oryzae* performed so far, and consider future possibilities for lipid production.

## 2 Research on lipid production using *A. oryzae*

Research on lipid production using *A. oryzae* is mainly categorized into two types: one that focuses on free fatty acid (FFA) production, and the other that focuses on ester-type fatty acid (EFA) production. EFAs correspond to FAs present in acylglycerols, such as triacylglycerol, in which FAs are fused to glycerol via an ester bond. Herein, I summarize research on FFA and EFA production using *A. oryzae*. In particular, I will explain FFA production that we have primarily studied in detail.

### 2.1 FFA production

Tamano et al. conducted a study on FFA production in *A. oryzae*. The results were categorized into three parts: FFA productivity enhancement, FFA secretory production, and FFA polyunsaturation. The details of this process are described below.

#### 2.1.1 Enhancement of FFA productivity

**Figure 1:**
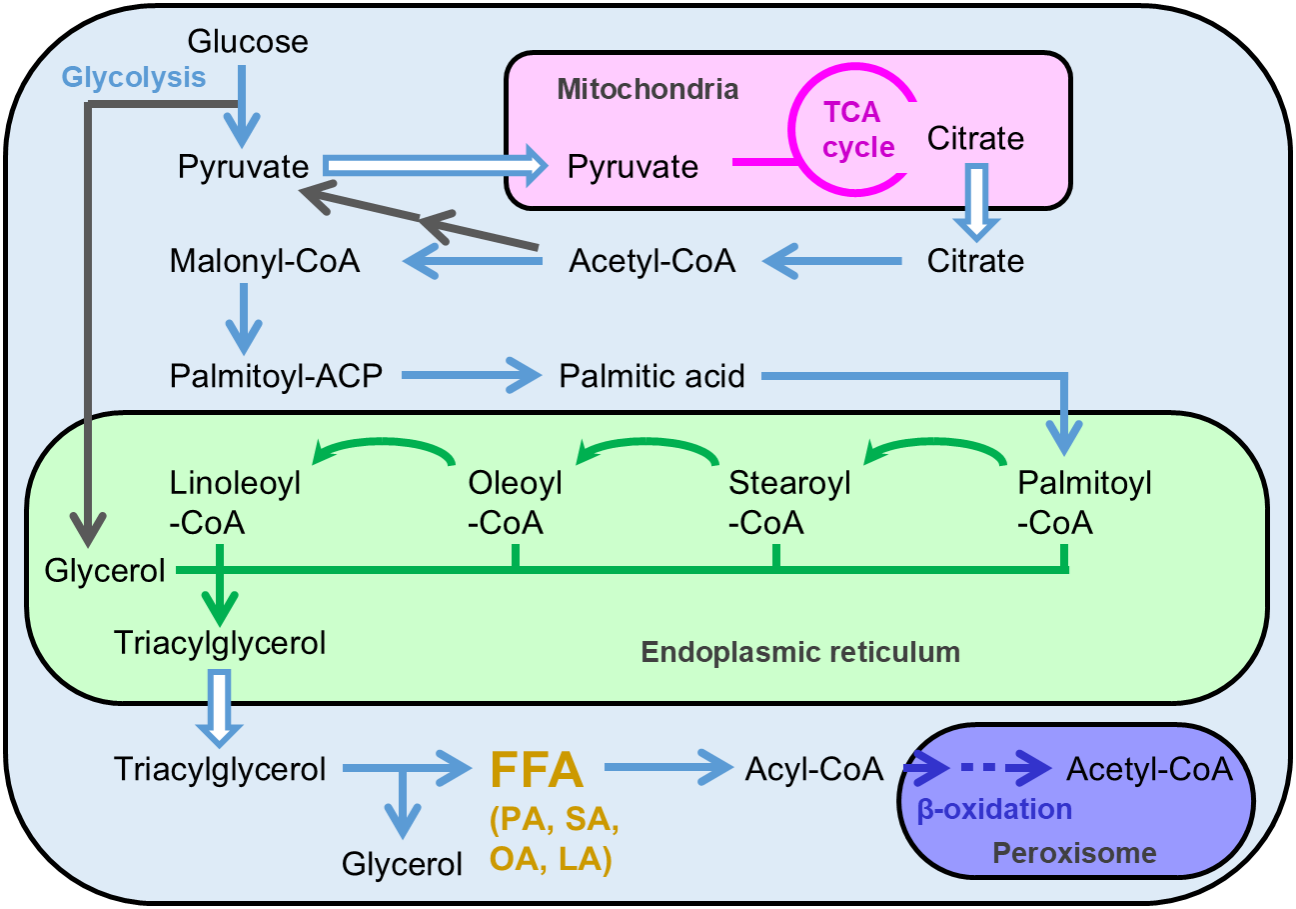
Presumable metabolic map of free fatty acid and triacylglycerol production in *Aspergillus oryzae*. FFA, free fatty acid; PA, palmitic acid; SA, stearic acid; OA, oleic acid; LA, linoleic acid.

*A. oryzae*’s FFA productivity was enhanced by improving the metabolic pathways involved in the FFA biosynthesis. It is considered that FFA biosynthesis is performed via the metabolic reactions illustrated in **Figure 1**. When *A. oryzae* is cultured in a liquid medium containing sufficient glucose (10%), citrate accumulates in the mitochondria via the catabolism of glucose during glycolysis and the TCA cycle. Consequently, citrate accumulated in the mitochondria is released into the cytosol and then converted to palmitic acid, a saturated C16 FFA, by a four-step enzyme reaction. Subsequently, palmitic acid is added to the CoA residue by an acyl-CoA synthetase and concomitantly transferred to the endoplasmic reticulum, where palmitic acid, as the CoA derivative, is modified by elongation and desaturation of the carbon chain. After modification, different fatty acyl-CoA molecules are fused to glycerol, leading to the generation of triacylglycerol. Triacylglycerol is released from the endoplasmic reticulum into the cytosol in the form of lipid droplets. When lipase interacts with triacylglycerol present in cytosol under conditions such as carbon-source starvation, triacylglycerol is degraded to FFA, followed by further degradation to acetyl-CoA via beta-oxidation.

**Figure 2:**
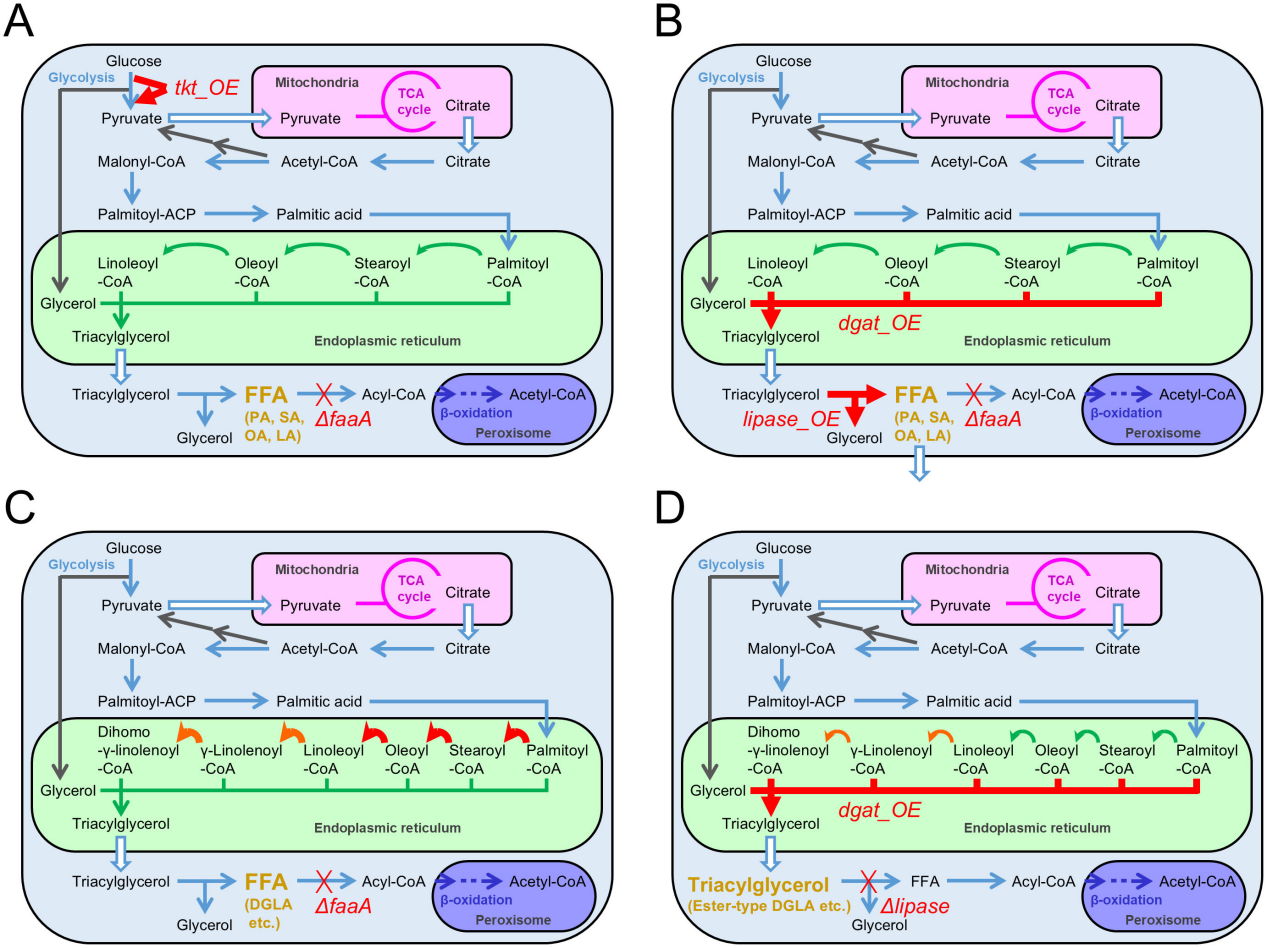
Metabolic modifications in each *Aspergillus oryzae* mutant constructed for increasing fatty acid productivities. Thick arrows indicate the metabolic reactions where the enzyme genes were overexpressed. Thick crosses indicate knockouts of the metabolic reactions. DGLA: dihomo-γ-linolenic acid. **(A)** Increase of free fatty acid productivity by *faaA* knockout and *tkt* overexpression. **(B)** Increase in free fatty acid secretory productivity by either overexpression of the *dgat* gene or the lipase gene with the *faaA* knockout in the presence of 1% Triton X-100. **(C)** Increased productivity of dihomo-γ-linolenic acid as a free fatty acid in the DGLA3 strain via the overexpression of endogenous genes involved in elongation or desaturation and by heterologous overexpression of the genes involved in the biosynthesis derived from *Mortierella alpina*. **(D)** Increased productivity of dihomo-γ-linolenic acid as an ester-type fatty acid in triacylglycerol via overexpression of the *dgat* gene and knockout of the lipase gene.

Firstly, FFA productivity was increased by FFA accumulation, which interrupted its degradation. For this purpose, we attempted to remove acyl-CoA synthetase that converts FFA to fatty acyl-CoA (**Figure 2A**). Six genes in *A. oryzae* RIB40 strain’s genome were highly homologous to the acyl-CoA synthetase genes identified in the budding yeast *Saccharomyces cerevisiae* by a BLASTP homology search. These six genes were individually knocked out in *A. oryzae* by genetic engineering, followed by an evaluation of FFA productivity, which is the amount of FFAs produced per gram of dried hyphae. Each strain was cultured in 50 mL Czapek–Dox minimal medium at 30°C and 200 rpm for 120 h, followed by extraction of FFAs in chloroform after disrupting cells and the subsequent application to the FFA enzyme assay. Among the six knockout mutants tested, only the mutant of the acyl-CoA synthetase gene *faaA* (AO090011000642), which has high homology to the acyl-CoA synthetase gene *FAA1* of *S. cerevisiae*, demonstrated a 9.2-fold increase in FFA productivity compared to the parental strain (Tamano et al., 2015).

Secondly, FFA productivity was further increased by strengthening FFA biosynthesis via metabolic improvement. NADPH is required as a cofactor for FFA biosynthesis by supplying a reducing force. Thus, more NADPH was considered necessary for *A. oryzae* to produce additional FFAs. Therefore, we focused on the pentose phosphate pathway in this study, which diverges from and merges to glycolysis like the bypass and consists of eight enzymatic reactions. This pathway provides pentose molecules as substrates for nucleic acids and generates NADPH. The genes responsible for the pentose phosphate pathway in *A. oryzae* were selected by referring to a study on the metabolic modeling of *A. oryzae* (Vongsangnak et al., 2008) and homology search results against the genes of pathways identified in other organisms. Ten genes were selected in total. Individual overexpression mutants of the selected genes were constructed using the *A. oryzae faaA* knockout mutant. Evaluation of the FFA productivity revealed that overexpression of the transketolase gene *tkt* (AO090023000345) in the *faaA* mutant increased FFA productivity 1.4-fold (**Figure 2A**) (Tamano and Miura, 2016). Taken together, combining *faaA* knockout and *tkt* overexpression increased FFA productivity 13-fold compared to the parental wild-type strain. Furthermore, the FFA production yield was attained at 2.7 g/L for a 5-day culture by complementing auxotrophy in the constructed mutant and enhancing nitrogen source concentration in the culture medium. This yield corresponded to a 90-fold increase from the original condition when the wild-type strain was cultured in a regular culture medium. Therefore, FFA yield was substantially increased via metabolic and culture improvements.

#### 2.1.2 FFA secretory production

Under regular culture conditions, *A. oryzae* accumulates FFAs within the cells, requiring cell disruption for extraction of FFAs with the aim of their industrial utilization. However, disrupting cells is labor-intensive, not eco-friendly, and requires mechanical cell treatment such as bead beating or chemical cell treatment using organic solvents such as acetone. Cell disruption may become unnecessary if FFAs are released from the cells. Therefore, FFA extracellular release, namely FFA secretory production, was attempted in *A. oryzae*.

To induce FFA extracellular release from *A. oryzae*, it was hypothesized that FFAs would be released if the cell surfaces were injured by chemicals or materials. Under these assumptions, different culture conditions were tested for the *A. oryzae faaA* knockout mutant. When Triton X-100, a non-ionic surfactant, was added to the liquid medium at a final concentration of 1%, the *faaA* mutant grew healthily and sufficiently, and FFAs were released to culture supernatant (Tamano et al., 2017). The amount of released FFAs corresponded to over 80% of the total FFAs produced. Moreover, extracellular release was limited to FFA and the alkyl ester. Another major lipid, triacylglycerol, which is mainly contained in lipid droplets, was not released. Furthermore, release was confirmed in the wild-type parental strain of *A. oryzae*. Taken together, FFA extracellular release was likely due to the increased permeability of cell membranes and protection of cells from bursting with endogenous rigid cell wall structures. Moreover, triacylglycerol was not released possibly because its large macromolecular lipid droplets cannot pass through the pores of the membranes created by Triton X-100.

Additionally, the enhancement of the productivities of released FFAs was studied under the culture condition with 1% Triton X-100 supplementation. Herein, genes that demonstrated positive correlations between expression levels and released FFA amounts in the time-course shifts, and the identified or predicted functions relative to lipid metabolism were selected. Subsequently, the selected genes were comprehensively overexpressed in the *faaA* knockout mutant. Overexpression of a lipase gene (AO090701000644) involved in generating FFAs and overexpression of a diacylglycerol O-acyltransferase gene *dgat* (AO090011000863) involved in generating triacylglycerol, which is a lipase substrate, had almost the same effect on enhancing FFA secretory productivity (**Figure 2B**) (Wong et al., 2021). The *faaA* mutant, which individually overexpressed these genes, demonstrated an approximately 3-fold increase in FFA secretory productivity in the presence of 1% Triton X-100.

#### 2.1.3 FFA polyunsaturation

Polyunsaturation of FFAs was also attempted. The wild-type RIB40 strain of *A. oryzae* originally biosynthesizes four FFAs (palmitic acid, stearic acid, oleic acid, and linoleic acid). However, FFAs expected to be used as feedstock for pharmaceutical agents, and supplements are more polyunsaturated. Such polyunsaturated FFAs include docosahexaenoic, eicosapentaenoic, and arachidonic acids. Thus, FFA polyunsaturation produced in the *A. oryzae faaA* knockout mutant was attempted. For this purpose, Δ6-desaturase and Δ6-elongase genes derived from *M. alpina* were introduced into the *A. oryzae faaA* mutant in a heterologous expression manner (**Figure 2C**). The introduced strain successfully biosynthesized free dihomo-γ-linolenic acid. That is, Δ6-desaturase and Δ6-elongase functioned to convert linoleic acid to dihomo-γ-linolenic acid in *A. oryzae* (Tamano et al., 2019). The constructed strain was named DGLA1.

Subsequently, overexpression of the three enzyme genes encoding endogenous elongase (AO090003000572), Δ9-desaturase (AO090011000488), and Δ12-desaturase (AO090001000224) were applied to the DGLA1 strain for facilitating the conversion of palmitic acid to linoleic acid. The multiple overexpression mutant of these three genes was named DGLA3 (**Figure 2C**). The DGLA3 strain produced 1.8-fold higher levels of free dihomo-γ-linolenic acid than the DGLA1 strain. The production yield reached 284 mg/L in the liquid culture (Tamano et al., 2020).

Furthermore, the *tkt* gene overexpression as described in section 2.1.1 was introduced to the DGLA3 strain, which increased free dihomo-γ-linolenic acid productivity by 1.2-fold according to the increase in the total FFA productivity. Subsequently, knocking out the α-1,3-glucan synthase gene *agsB* (AO090003001500) involved in pellet formation of *A. oryzae* (Zhang et al., 2017b) further enhanced free dihomo-γ-linolenic acid productivity by 1.1-fold. This finally constructed strain called DGLA3_tktOE_ΔagsB produced 533 mg of free dihomo-γ-linolenic acid per liter of liquid culture (Tamano et al., 2023). Overexpression of *tkt* and knockout of *agsB* led to dispersed hyphae in the liquid culture. This improved efficiencies of glucose and oxygen uptakes compared to the original pellet form, thus enhancing productivity. Additionally, the production yield of free dihomo-γ-linolenic acid increased more than the productivity. This was attributed to the increase in biomass owing to the shift from pellet to dispersed hyphae. In other words, hyphal density increased by dispersion, contributing to an increase in production yield.

### 2.2 EFA production

EFA incorporated into triacylglycerol has also been studied for their production in *A. oryzae*. Research on the production of valuable FAs in the EFA form and on increasing the production yield by metabolic improvement was performed by Laoteng et al. using the *A. oryzae* wild-type strain BCC7051 found in Thailand as a parental host. They first introduced the Δ6-desaturase and Δ6-elongase genes from *Pythium* sp. into BCC7051 in a heterologous manner. Consequently, dihomo-γ-linolenic acid was produced in an EFA form from linoleic acid (Chutrakul et al., 2016). Subsequently, they sequenced the BCC7051 genome, referred to the genomic information, and overexpressed the endogenous diacylglycerol O-acyltransferase gene *dgat* in the mutant (**Figure 2D**). Thus, the production yield of the ester-type dihomo-γ-linolenic acid could be enhanced. Moreover, in the BCC7051 strain, two endogenous lipase genes were found to be involved in acylglycerol degradation. They subsequently knocked them out and constructed single- and duplicate-knockout mutants via recombination (**Figure 2D**). Both single and duplicate mutants demonstrated increased triacylglycerol production (Anantayanon et al., 2021).

## 3 Discussion

I concisely summarized the advancements of lipid production research performed so far using *A. oryzae* as abovementioned. The productivity of FAs could successfully be increased by metabolic improvement based on the metabolic map and/or transcription profile. There, a metabolic reaction considered unnecessary for target metabolite’s biosynthesis was knocked out, whereas metabolic reaction considered as a bottleneck for the biosynthesis was overexpressed.

To further enhance the production of valuable target metabolites, such as FAs, using *A. oryzae*, utilizing flux balance analysis with the metabolic model to optimize the metabolism for target metabolite production will be meaningful. Additionally, metabolomics can be used to comprehensively measure precursors of a target metabolite via mass spectrometry, identifying a bottleneck reaction in biosynthesis. Genetic engineering can then be employed to overexpress the enzyme gene responsible for the bottleneck. Currently, these analyses and measurements are limited to specialized research groups with expertise in bioinformatics and access to expensive mass spectrometry equipment. However, as mass spectrometry devices prevail with the development of user-friendly software for flux balance analysis, it is expected that these techniques will be used more frequently to boost production yields.

Compared to model microorganisms such as *Escherichia coli* and *S. cerevisiae*, *A. oryzae* has some disadvantages: i) slower colony growth rate, ii) unavailability of breeding via mating, and iii) the necessity of single-spore isolation to construct mutants because of the cell’s multinuclear characteristics. However, *A. oryzae* is advantageous in that it has high metabolic activity and produces large amounts of secretory hydrolases such as amylase and protease. Moreover, *A. oryzae* has a DNA splicing function specific to eukaryotic cells; therefore, it is deemed more suitable as a host for producing metabolites derived from eukaryotes, such as plants, mushrooms, and fungi, than prokaryotic microorganisms. Furthermore, *A. oryzae* does not produce endogenous secondary metabolites, except kojic acid (Terabayashi et al., 2010), which is considered advantageous for purifying valuable metabolites produced in a heterologous manner because of low quantities of endogenous secondary metabolites. While challenges remain, there is hope that *A. oryzae* will see increased industrial use for producing valuable metabolites, including FAs, in the future.
